# Exploring efficient and effective mammalian models for Alzheimer’s disease

**DOI:** 10.3389/fnagi.2025.1652754

**Published:** 2025-08-14

**Authors:** Mitsunori Kayano

**Affiliations:** Research Center for Global Agromedicine, Obihiro University of Agriculture and Veterinary Medicine, Obihiro, Japan

**Keywords:** amyloid-β, α-synuclein, blood brain barrier, cerebral amyloid angiopathy, marmoset, dog, tree shrew, rodent

## Abstract

The aim of this study was to explore and discuss efficient and effective mammalian models for Alzheimer’s disease (AD). In this study, efficient AD models are characterized by a small body size, a short lifespan, and rapid development of the main pathology including amyloid plaque formation. Effective AD models are expected to exhibit not only the main pathology, but also co-pathology associated with other neurodegenerative diseases (e.g., Lewy body dementia), systemic disturbances such as disrupted central–peripheral homeostasis, and sleep-circadian failures. This reflects recent findings indicating that AD is far more multifactorial than previously assumed. Although further investigation is required, non-human primates, particularly common marmosets (*Callithrix jacchus*), and dogs (*Canis lupus familiaris*) are candidates of promising and effective AD models. Tree shrews (*Tupaia belangeri*), guinea pigs (*Cavia porcellus*), and evolutionary related species including degus (*Octodon degus*) constitute an alternative group of AD models that remain underexplored but potentially efficient and effective. These mammalian models, together with hypothesis-driven mouse models and advances in data science technologies including omics and imaging analyses, may lead to breakthroughs in AD research, resulting in the development of effective prevention and treatment for AD.

## 1 Introduction

Alzheimer’s disease (AD), the most common form of dementia, is characterized by the presence of β-amyloid (Aβ)-containing extracellular plaques and tau-containing intracellular neurofibrillary tangles in the brain (Hardy and Selkoe, 2002; Bloom, 2014; Scheltens et al., 2021). In amyloid hypotheses, form(s) of Aβ, such as plaques and soluble oligomers, in the brain initiates a pathophysiological cascade leading the tau pathology, neuro-inflammation related to activation of microglia and astrocyte, neuronal death and cognitive decline (Selkoe and Hardy, 2016; Cline et al., 2018). However, prevention and treatment targeting the brain Aβ have not been as successful as the amyloid hypotheses had expected.

AD may be far more multifactorial than previously assumed, regarding co-pathology and systemic abnormalities. For example, emerging evidences have supported that AD brains frequently share the pathology (co-pathology) associated with other dementias such as Lewy body dementia (LBD) and frontotemporal lobe dementia (FTLD) through the interplay among Aβ, tau, α-synuclein and TAR DNA-binding protein of 43 kDa (TDP-43) (Robinson et al., 2018; Sengupta and Kayed, 2022). Also, AD may extend beyond the brain, involving systemic alterations (Wang et al., 2017; Cheng et al., 2020; Xu et al., 2025). At least 10 to 20 multilevel factors at molecular, cellular, tissue-organ and individual levels are then associated with AD: (1) molecular level: Aβ, tau, α-synuclein, TDP-43 and apolipoprotein E (APOE; a major risk factor for AD) (Yamazaki et al., 2019; Serrano-Pozo et al., 2021; Jackson et al., 2024), (2) cellular level: astrocyte, microglia, oligodendrocyte, T-cell and neutrophil (Huang et al., 2022; Gericke et al., 2023), (3) tissue-organ level: cortex, hippocampus, hypothalamus, liver, pancreas, kidney and gut (Wang et al., 2017; Xu et al., 2025), and (4) individual level: infection (Moir et al., 2018; Vojtechova et al., 2022), sleep-circadian failure (Ju et al., 2014; van Erum et al., 2018), cardiovascular diseases (Stampfer, 2006; Tini et al., 2020), diabetes (Sims-Robinson et al., 2010; Takeda et al., 2010; Arnold et al., 2018) and epilepsy (Palop and Mucke, 2009; Horváth et al., 2016).

**FIGURE 1:**
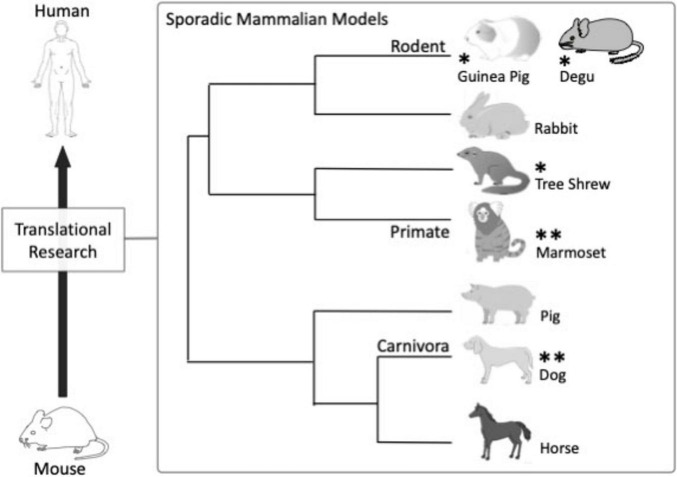
Divergence of sporadic mammalian models for the multifactorial AD. Non-human primates, particularly common marmosets, and dogs can be promising and effective animal models for the multifactorial AD. Tree shrews, guinea pigs and degus constitute an alternative group of AD models that remain underexplored but potentially efficient and effective. Surprising fact is that dog, a mammalian species which is evolutionally divergent from human, can naturally develop human-like AD without any interventions. This supports the use of naturally occurring animal models for AD. The possibility of large and small mammals including pig, cattle, horse, rabbit, ferret, Mongolian gerbil as the AD model is discussed in the Supplementary material. **, promising and effective models (marmosets and dogs); *, underexplored but potentially efficient and effective models (tree shrews, guinea pigs and degus).

Interestingly, many mammalian species spontaneously exhibit the amyloid plaque as they age (Mckean et al., 2021; Sharma et al., 2023; Ferrer, 2024; Supplementary material). Several mammalian species also naturally present the tau pathology (Mckean et al., 2021) and some show symptoms as well (Osella et al., 2007; Prpar Mihevc and Majdič, 2019). The most surprising fact is that dogs, a mammalian species which is evolutionally divergent from human in mammals (Figure 1), can naturally develop AD-like disorder without any interventions: it is canine cognitive dysfunction (CCD) (Osella et al., 2007; Landsberg et al., 2017; Prpar Mihevc and Majdič, 2019). CCD dogs can exhibit the amyloid plaque and tau pathology, and, very surprisingly, they present symptoms exactly like human such as disorientation (Osella et al., 2007). This fact encourages us and evokes idea of naturally onset mammals for AD, implying that key progression pathways of the multifactorial AD may be fundamentally conserved in mammals.

This review provides the summary of the possibility of effective mammalian models for the multifactorial AD. Also, the efficiency including a small body size, a short life span and rapid disease progression of the animals is discussed.

## 2 Key criteria for efficient and effective animal models for Alzheimer’s disease

Animal models are expected to be efficient and effective. For the efficiency, the AD models need to have a small body size (low maintenance costs), a short lifespan (short generation time) and rapid disease progression: models are particularly required to exhibit the Aβ accumulation and plaques as early as possible with preserved effectiveness.

For the effectiveness, animal models for AD are expected to exhibit at least the amyloid and tau pathology, and also co-pathology such as those involving α-synuclein and systemic alterations including direct dysfunction in peripheral tissues, breakdown of blood-brain-barrier (BBB) and disruption of central and peripheral homeostasis. This reflects recent findings indicating that AD is far more multifactorial than previously assumed, involving co-pathology and systemic alterations. Although α-synuclein can be detected in the brain, particularly as a component of the amyloid plaque (Ueda et al., 1993), it can be detected in peripheral, particularly in gut (Xu et al., 2025). This may construct the gut-to-brain axis of α-synuclein spreading. AD patients also show systemic alterations. Typical systemic alterations associated with AD include the Aβ and tau accumulations in peripheral tissues and organs (Xu et al., 2025), peripheral inflammation and dysfunction (Wang et al., 2017) and breakdown of BBB (Bowman et al., 2007; Sweeney et al., 2018; Cai et al., 2018). Especially, BBB impairment is thought to be critical in the multifactorial AD: since BBB maintains physiological and immunological homeostasis in central nervus system (CNS) and periphery, and BBB leakage may precede the senile plaque (Ujiie et al., 2003), implying that BBB alterations may be linked to the true initiator(s) of AD. BBB dysfunction is believed to be associated with cerebral amyloid angiopathy (CAA) (Kalaria, 1999; Carrano et al., 2012; Magaki et al., 2018) and epilepsy (van Vliet et al., 2007; Löscher and Friedman, 2020; Greene et al., 2022). APOE may also associate with BBB dysfunction (Montagne et al., 2020; Liu et al., 2022). Finally, sleep and circadian disorders are probably associated with huge numbers of factors in the multifactorial AD including co-pathology and systemic alterations (Ju et al., 2014; Musiek and Holtzman, 2016; Cuddapah et al., 2019; Patke et al., 2020; Nassan and Videnovic, 2022). We need to sleep for our health (Buysse, 2014; Mander et al., 2017): not only for AD, but also for other neurodegenerative diseases (Malhotra, 2018; Husain, 2021).

In summary, for the efficiency, the models are required to exhibit the amyloid and related pathology as early as possible. In addition, a smaller body size is preferred to minimize maintenance costs. The effective models for the multifactorial AD should exhibit at least the amyloid and tau pathology. Also, co-pathology of α-synuclein and related accumulation in the brain and body are expected to be observed. For the systemic abnormalities in AD, the models need to present direct alterations in peripheral tissues, BBB dysfunction and/or related disorders such as CAA and epilepsy that are associated with disrupted peripheral-central homeostasis. Moreover, sleep-circadian failures, which are related to co-pathology and systemic abnormalities, are desirable features to be observed. However, it seems impossible to obtain detailed studies for all aspects of co-pathology, systemic alterations and sleep-circadian failures in underexplored animals. This study then evaluates the effective model as the possibility of either co-pathology or at least one of the systemic alterations, and roughly discusses (1) whether each animal species is diurnal and (2) selective sleep-circadian reports in each species.

## 3 Efficient and effective mammalian models for Alzheimer’s disease

### 3.1 Non-human primate: common marmosets

Non-human primates (NHPs) including common marmosets (marmoset; *Callithrix jacchus*) are probably promising and effective models for AD (Mckean et al., 2021; Stonebarger et al., 2021; Rizzo et al., 2023). In particular, marmosets, a small primate species (∼300 g body weight in wild), are an emerging model for AD and other neurodegenerative diseases (Rizzo et al., 2023; Pérez-Cruz and Rodríguez-Callejas, 2023; Huhe et al., 2025). Marmosets are considered to be aged in 8–10 years and have average lifespan of 12 years (Table 1) (Pérez-Cruz and Rodríguez-Callejas, 2023). Sporadic amyloid plaques are observable as early as 7 years (Rizzo et al., 2023), and aged marmosets exhibit both 3-repeat (3R) and 4-repeat (4R) tau isoforms in their brain, implying highly toxic patterns of tau expressions similar to human (Huhe et al., 2025). Aggregation of α-synuclein in the marmoset brain and body is thought to be possible (Shimozawa et al., 2017): the natural aggregation of α-synuclein in the olfactory bulb of 6 years old marmoset (without injections of toxic seeds) has been reported (Kobayashi et al., 2016). Also, colitis may be associated with the alteration in α-synuclein expression and phosphorylation in the myenteric plexus of marmosets (Resnikoff et al., 2019). CCA pathology (Rizzo et al., 2023) and epilepsy (Yang et al., 2022) which are associated with BBB disruption are naturally observed in marmosets: direct BBB characteristics of marmosets have also been well-studied (Hoshi et al., 2013; Parks et al., 2023). The marmoset is diurnal, and the sleep and circadian rhythm of marmosets has been well-studied (Erkert, 1989; Crofts et al., 2001; Koshiba et al., 2021; Bukhtiyarova et al., 2022).

**TABLE 1:** Comparison of human and selective mammalian models for efficient and effective Alzheimer’s disease (AD) research.

Species		Body size*^1^	Lifespan (y: years)	Plaque (y: years)	Tau	Alpha-synuclein	Systemic abnormalities*^2^	Diurnal
Human	(*Homo sapience*)	Large	80 y	> 50–60 y	Yes	Yes	Yes	Yes
Marmoset	(*Callithrix jacchus*)	Small	12 y	> 7–10 y	Yes	Probable	Probable	Yes
Dog	(*Canis lupus familiaris*)	Middle	15 y	> 8–10 y	Yes	Probable	Probable	Yes
Tree shrew	(*Tupaia belangeri*)	Small	6–8 y	> 5–6 y*^3^	Yes	Probable	Possible	Yes
Guinea pig	(*Cavia porcellus*)	Small	5–7 y	> 4 y	Yes	Possible	Probable	Yes
Degu	(*Octodon degus*)	Small	5–7 y	> 3 y*^4^	Yes	ND	Probable	Possible
Rat	(*Rattus norvegicus*)	Small	2 y	ND	ND	ND	Possible*^5^	No
Mouse	(*Mus musculus*)	Tiny	2 y	ND	ND	ND	Possible*^5^	No

Body size, lifespan and the timing of plaque deposition are associated with the efficiency. Others are related to the effectiveness for the multifactorial Alzheimer’s disease (AD) regarding the main pathology (plaque and tau), co-pathology, systemic alterations and diurnality. Yes/No/Probable/Possible is determined by their natural and wild situation, at most with diet/metal/environmental supplementation (without any drug-induced and/or genetic manipulation). ND means “not detected.”

*1 Body size: tiny (< 100 g), small (< 1 kg), middle (< 30 kg), and large (> 30 kg).

*2 “Probable/Possible” refers possibility of direct alterations in peripheral tissues, blood-brain-barrier (BBB) disruption and related diseases such as cerebral amyloid angiopathy (CAA) and epilepsy that are associated with disrupted peripheral-central homeostasis.

*3 In tree shrews, the presence of the amyloid plaque is thought to be relatively rare.

*4 Degus with APOE4 allele and wild-captured or early generations after the wild-capture may frequently develop the amyloid pathology.

*5 BBB characteristic and dysfunction with aging have been studied in mice (Poduslo et al., 2001; Daneman et al., 2010; Braniste et al., 2014; Goodall et al., 2018) and rats (Yang et al., 1994; Enerson and Drewes, 2006; Hawkins et al., 2007).

### 3.2 Companion animal (divergent from primates): dogs

Dogs, a Carnivora species, are another candidate of promising and effective models for AD (Cotman and Head, 2008; Prpar Mihevc and Majdič, 2019; Ambrosini et al., 2019). Dog is evolutionally divergent from human in mammals (more distant than rodents; Figure 1), but can naturally develop AD-like disorder without any interventions. Sever cognitive decline can be observed in aged dog: it is referred as CCD. Aged dogs can naturally exhibit both amyloid plaque (over 8 to 10 years-old) and tau pathology (Schmidt et al., 2015; Ozawa et al., 2016; Smolek et al., 2016; Table 1), and, very surprisingly, present symptoms exactly like human such as disorientation (Osella et al., 2007). Accumulation of α-synuclein has been presented in spinal cord and hippocampus of 10–12 years old beagle dogs (Ahn et al., 2012; Ahn et al., 2013), although it may be not very frequent and/or breed-genetical specific (Uchida et al., 2003). CAA including micro-bleeding in the brain and epilepsy (probable BBB disruption) seems relatively frequent in aged dogs (Wisniewski et al., 1996; Ozawa et al., 2016; Nešić et al., 2017). Direct association studies between epilepsy and BBB disfunction in dogs have been reported (Hanael et al., 2019; Hanael et al., 2024). Sleep-circadian cycle has been widely studied in dogs (Adams and Johnson, 1993; Bódizs et al., 2020; Reicher et al., 2021).

### 3.3 Close to primates: tree shrews

Tree shrews (northern tree shrew; *Tupaia belangeri*), a species in the order Scandentia and widely distributed in South and Southeast Asia, are a possible efficient and effective model for AD (Fan et al., 2018; Li et al., 2023). The advantages of tree shrew as model animals are a small body weight (100–150 g), a short lifespan (6–8 years) and low maintenance costs (Table 1). Tree shrew has a much closer genetic and physiological affinities to primates than those of rodents (Figure 1) and has been used as models for basic science and many types of diseases including brain development, infection (particularly hepatitis viruses), depression, social stress and aging (Fan et al., 2018; Li et al., 2023; Yao et al., 2024). Aβ aggregates, plaque-like structures and increased phosphorylated tau protein have been detected in the brain of 5–6 years-old tree shrews (Yamashita et al., 2012; Fan et al., 2018; Li et al., 2023), although the plaque deposition may be rare (Pawlik et al., 1999; Yamashita et al., 2010; Li et al., 2023). The α-synuclein protein sequence of tree threw is 97.1% identical to that of human, implying the tree shrew’s α-synuclein might have similar functions compared to human (Wu et al., 2015). A higher expression and aggregates of α-synuclein has been observed in the brain of tree shrews (Wu et al., 2019). Although CAA, epilepsy and related BBB leakages have not been reported, gut-to-brain axis in cognition (Guo et al., 2021; Wang et al., 2023) and circadian rhythm (Meijer et al., 1990; Legros et al., 2007; Coolen et al., 2012; Luo et al., 2020; Dimanico et al., 2021) of tree shrews has been studied well.

### 3.4 Rodents: guinea pigs and degus

Rodents other than mice and rats can be other candidates of the efficient and effective models for AD. The order Rodentia (rodents) is divided into three suborders: Sciuromorpha (squirrel-like) and Myomorpha (mouse and rat-like), and Hystricomorph (porcupine-like). In particular, certain hystricomorph rodents, including guinea pigs (*Cavia porcellus*: Sharman et al., 2013; Wahl et al., 2022) and degus (*Octodon degus*: Inestrosa et al., 2005; Hurley et al., 2018), are emerging AD model rodents. A relatively smaller body size and a shorter lifespan of such rodents than other mammalian models suggest their potential as one of the most efficient models for AD. Moreover, such rodents can be effective and are expected to bridge the translational gap between mouse to human, since they are rodents like mice and rats, but have potential to naturally onset AD.

The guinea pig, a hystricomorph rodent with an average lifespan of 5–7 years and a body weight of 700 to 1,000 g, is an emerging sporadic model for AD (Sharman et al., 2013; Wahl et al., 2022; Table 1). Guinea pigs have been used in research for over 200 years (Harkness et al., 2002), including more recent studies of cerebral cortices (Hatakeyama et al., 2017), infectious diseases (Connolly et al., 1999; Lowen et al., 2006; Padilla-Carlin et al., 2008) and pharmacological, environmental, and dietary interventions (Rakic et al., 1989; Kim et al., 2017; Li et al., 2021). Guinea pigs have the identical Aβ_42_ sequence to human (Salazar et al., 2016) and express both 3R and 4R tau isoforms (Sharman et al., 2013). The Aβ aggregates (Wahl et al., 2022) and plaques (Bates et al., 2014) can be observed at over 1 and 4 years old, respectively. Although this study has not found any report of the aggregates and/or deposition of α-synuclein in the brain of guinea pigs, the aggregation has been observed in the gut (Sharrad et al., 2013; Sharrad et al., 2017): notice that two possible pathways (brain-first and body (gut)-first) for the synuclein spreading have been reported (Borghammer and Van Den Berge, 2019; Nuzum et al., 2022). The BBB of guinea pigs has been well-studied (Rakic et al., 1989; Uva et al., 2008), including the transport of Aβ at BBB (Martel et al., 1996). No CAA researches were found in this study. Pharmacologically-induced epilepsy in guinea pig have been widely investigated, and the association between epilepsy and BBB permeability using induced-epilepsy model of the guinea pig has been studied (Uva et al., 2008). Also a gut-to-brain (microbiome-hypothalamus) axis in guinea pigs has been investigated (Li et al., 2021; Nuzum et al., 2022). Guinea pigs are diurnal, and extensive studies have been reported related to sleep and circadian system in guinea pigs (Kurumiya and Kawamura, 1988; Akita et al., 2001; Liu et al., 2020).

The degu, another hystricomorph rodent from central Chile with an average lifespan of 5 to 7 years and a body weight of less than 300 g, has the potential as one of the most efficient and effective mammalian models for AD (Inestrosa et al., 2005; Cisternas et al., 2018; Hurley et al., 2018; Tan et al., 2022; Table 1). Degus are highly social (Rivera et al., 2016) and thought to have advanced cognitive abilities (Kumazawa-Manita et al., 2013), although they are a small rodent. Degus are the emerging candidate of multimorbidity-systemic models, since recent studies have reported that degus naturally develop visual impairments (Datiles and Fukui, 1989; Szabadfi et al., 2015; Hurley et al., 2018), endocrinological and metabolic dysfunctions including diabetes (Datiles and Fukui, 1989; Rivera et al., 2018; Hurley et al., 2018) and neoplasi (Anderson et al., 1990; Lester et al., 2005; Švara et al., 2020; Ikeda et al., 2024). Interestingly, related to AD pathology, degus spontaneously represent the accumulation of Aβ and phosphorylated tau in the brain: it may start between 1 and 3 years old (Ardiles et al., 2012). The amyloid plaque can be observed at 3–5 years old (Cisternas et al., 2018). Although some studies have reported contradictory results regarding the potential of degus as a model for AD research (Steffen et al., 2016; Bourdenx et al., 2017), degus with the higher risk APOE4 allele and wild-captured or early generations after the wild-capture may frequently develop the AD pathology (Hurley et al., 2022). No α-synuclein researches in degus were found in this study. However, degus can be one of the best (efficient and effective) models for investigating systemic alterations in AD, because they can naturally exhibit CAA (van Groen et al., 2011) and epilepsy (Ikai et al., 2021) (like marmosets and dogs), implying a higher risk for BBB dysfunction. Degus can be diurnal (Kas and Edgar, 1999a; Lee, 2004; Bonmati-Carrion et al., 2017), and sleep and circadian functions including the association between sleep deprivation and cognitive decline have been studied well (Kas and Edgar, 1999b; Ocampo-Garcés et al., 2013; Tarragon et al., 2014; Estrada et al., 2015).

## 4 Conclusion and perspective

Interestingly, many mammalian species spontaneously exhibit the amyloid plaques as they age (Mckean et al., 2021; Sharma et al., 2023; Ferrer, 2024; Supplementary material). This study updated the information of mammalian models for the multifactorial AD, including co-pathology and systemic alterations. However, it was impossible to investigate prevalence and frequency of such new issues for relatively underexplored animals. This study was based on case reports of mammals. This is a limitation of this review.

Although further investigation is required, NHPs, particularly marmosets, and dogs are candidates of promising and effective AD models. Previous studies have showed that marmosets and dogs can present the amyloid plaque at 7–10 years old. Tree shrews, guinea pigs and degus constitute an alternative group of AD models that remain underexplored but potentially efficient and effective. Particularly, in guinea pigs and degus, the amyloid plaque can be detected in 3–4 years: this seems the earliest (the most efficient) so far in naturally onset mammals. However the possibility of co-pathology and systemic alterations in the three species warrants further investigation for more robust and effective AD models.

Emerging evidences suggest that the key progression pathways of the multifactorial AD may be fundamentally conserved in several mammalian species. The efficient and effective mammalian model provides opportunities to investigate the long-term and spatio (systemic)-temporal observations that are potentially crucial in current AD research but are highly resource-intensive and time-consuming to implement in epidemiological studies of NHPs (except marmosets) and human. The mammalian models in this study, together with hypothesis-driven mouse models and also advances in data science technologies including omics and imaging analyses, may bridge the translational gap between mouse and human and lead to breakthroughs in AD research.
