# Supplementary material for: Exploring efficient and effective mammalian models for Alzheimer’s disease
Source: Front Aging Neurosci. 2025 Aug 14;17:1652754. doi: 10.3389/fnagi.2025.1652754 (PMC12391168; doi:10.3389/fnagi.2025.1652754)
Supplement: Supplementary file 1 [file Data_Sheet_1.pdf]

Supplementary material of

“Exploring efficient and effective mammalian models for Alzheimer’s disease”

Other possible models for the multifactorial AD: large and other small mammals

Other mammalian models for AD include 1) large mammals such as pigs, horses and cattle, and 2) small mammals including rabbits, ferrets and Mongolian gerbils. Although the large mammals may not be efficient, they have the potential as effective models (Eaton and Wishart, 2017), because some large mammals have presented spontaneous AD-like pathology involving amyloid plaques (Capucchio et al., 2010; Moreno-Gonzalez et al., 2022; Ferrer, 2024). Surprisingly, regarding the co-pathology, spontaneous increase of α-synuclein have been reported in the horse and cattle (McFarlane et al., 2005; Niyonzima et al., 2025). In the small mammals, rabbits (Sparks and Schreurs, 2003; Bitel et al., 2012; Weiss et al., 2022) can be one of the best candidates of efficient and effective AD models, since, for the effectiveness, diet and metal induced AD pathology (Sparks and Schreurs, 2003; Weiss et al., 2022) and disruption of BBB (Hathaway et al., 1999; Chen et al., 2008) in the rabbit have been studied. Ferrets have been used in the research of cortical folding and neuroscience (Gilardi and Kalebic, 2021; Ross et al., 2024), implying the effective translational studies for cognition and AD (Paulsson et al., 2011; Schwerin et al., 2021). Mongolian gerbils, a rodent species in the suborder of Myomorpha (mouse and rat-like), would be an efficient and effective model particularly for the systemic investigation in the multifactorial AD. Mongolian gerbils are tiny (~50g body weight) and have a 3 to 5 years life span. Also, they are one of the best models for spontaneous epilepsy (Lomax et al., 1986; Cutler and Mackintosh, 1989; Bertorelli et al., 1995), implying BBB disruption may be observed. However, compared to the mammals in the main text (**Table 1**), further investigations are required for such large and small mammals for AD research.

References

Bertorelli, R., Adami, M., and Ongini, E. (1995) The Mongolian gerbil in experimental epilepsy. *Ital. J. Neurol. Sci.* 16:101–106. doi:10.1007/BF02229081.

Bitel, C. L., Kasinathan, C., Kaswala, R. H., Klein, W. L., and Frederikse, P. H. (2012) Amyloid-β and tau pathology of Alzheimer’s disease induced by diabetes in a rabbit animal model. *J. Alzheimers Dis.* 32:291–305. doi:10.3233/JAD-2012-120571.

Capucchio, M. T., Márquez, M., Pregel, P., Foradada, L., Bravo, M., Mattutino, G., et al. (2010) Parenchymal and vascular lesions in ageing equine brains: histological and immunohistochemical studies. *J. Comp. Pathol.* 142:61–73. doi:10.1016/j.jcpa.2009.07.007.

Chen, X., Gawryluk, J. W., Wagener, J. F., Ghribi, O., and Geiger, J. D. (2008) Caffeine blocks disruption of blood–brain barrier in a rabbit model of Alzheimer’s disease. *J. Neuroinflamm.* 5:12. doi:10.1186/1742-2094-5-12.

Cutler, M. G. and Mackintosh, J. H. (1989) Epilepsy and behaviour of the Mongolian gerbil: an ethological study. *Physiol. Behav.* 46:561–566. doi:10.1016/0031-9384(89)90333-8.

Eaton, S. and Wishart, T. (2017) Bridging the gap: large animal models in neurodegenerative research. *Mamm. Genome* 28:324–337. doi:10.1007/s00335-017-9687-6.

Ferrer, I. (2024) Alzheimer's disease neuropathological change in aged non-primate mammals. *Int. J. Mol. Sci.* 25:8118. doi:10.3390/ijms25158118.

Gilardi, C. and Kalebic, N. (2021) The ferret as a model system for neocortex development and evolution. *Front. Cell Dev. Biol.* 9: 661759. doi:10.3389/fcell.2021.661759.

Hathaway, C. A., Appleyard, C. B., Percy, W. H., and Williams, J. L. (1999) Experimental colitis increases blood-brain barrier permeability in rabbits. *Am. J. Physiol. Gastrointest. Liver Physiol.* 276:G1174–G1180. doi:10.1152/ajpgi.1999.276.5.G1174.

Lomax, P., Lee, R. J., and Olsen, R. W. (1986). "The spontaneously epileptic Mongolian gerbil", in *Neurotransmitters and Epilepsy*, ed. P. C. Jobe and H. E. Laird (Totowa, NJ: Humana Press), 41-56.

McFarlane, D., Dybdal, N., Donaldson, M. T., Miller, L., and Cribb, A. E. (2005) Nitration and increased α-synuclein expression associated with dopaminergic neurodegeneration in equine pituitary pars intermedia dysfunction. *J. Neuroendocrinol.* 17:73–80. doi:10.1111/j.1365-2826.2005.01277.x.

Moreno-Gonzalez, I., Edwards, G., Morales, R., Duran-Aniotz, C., Escobedo, G., Pumarola, M., et al. (2022) Aged cattle brain displays Alzheimer's disease-like pathology and promotes brain amyloidosis in a transgenic animal model. *Front. Aging Neurosci.* 13:815361. doi:10.3389/fnagi.2021.815361.

Niyonzima, Y. B., Asato, Y., Murakami, T., Kadokawa, H. (2025) Alpha-synuclein expression in anterior pituitary cells of aged cattle. *Domest. Anim. Endocrinol.* 92: 106936. doi:10.1016/j.domaniend.2025.106936.

Paulsson, J. F., Westermark, G. T., Aberrant, V., Peng, S., and Westermark, P. (2011) Ferret islet amyloid polypeptide (IAPP): characterization of in vitro and in vivo amyloidogenicity. *Amyloid* 18(4): 191–199. doi:10.3109/13506129.2011.627956.

Ross, G., Radtke-Schuller, S., and Frohlich, F. (2024) Ferret as a model system for studying the anatomy and function of the prefrontal cortex: A systematic review. *Neurosci. Biobehav. Rev.* 159: 105600. doi:10.1016/j.neubiorev.2024.105600.

Schwerin, S. C., Rizzi, G., Radtke-Schuller, S., Fröhlich, F., and Shih, A. Y. (2021) Expression of GFAP and tau following blast exposure in the cerebral cortex of ferrets. *J. Neuropathol. Exp. Neurol.* 80: 112–121. doi:10.1093/jnen/nlab001.

Sparks, D. L. and Schreurs, B. G. (2003) Trace amounts of copper in water induce β-amyloid plaques and learning deficits in a rabbit model of Alzheimer's disease. *Proc. Natl. Acad. Sci. U.S.A.* 100:11065–11069. doi:10.1073/pnas.1832769100.

Weiss, C., Bertolino, N., Procissi, D., Aleppo, G., Smith, Q. C., Viola, K. L., et al. (2022) Diet-induced Alzheimer's-like syndrome in the rabbit. *Alzheimers Dement. Transl. Res. Clin. Interv.* 8:e12241. doi:10.1002/trc2.12241
